# Engineering of Phycourobilin Synthase: PubS to a Two-Electron Reductase

**DOI:** 10.1093/pcp/pcae098

**Published:** 2024-09-04

**Authors:** Keita Miyake, Saya Iwata, Rei Narikawa

**Affiliations:** Department of General Systems Studies, Graduate School of Arts and Sciences, The University of Tokyo, 3-8-1 Komaba, Meguro, Tokyo, 153-8902 Japan; Department of Biological Science, Faculty of Science, Shizuoka University, 836 Ohya, Sumga-ku, Shizuoka, 422-8529 Japan; Department of Biological Sciences, Graduate School of Science, Tokyo Metropolitan University, 1-1 Minami-Ohsawa, Hachioji, Tokyo, 192-0397 Japan

**Keywords:** Ferredoxin-dependent bilin reductase, Linear tetrapyrrole, Substrate-binding mode

## Abstract

**Phycourobilin:ferredoxin oxidoreductase (PubS) belongs to the ferredoxin-dependent bilin reductase (FDBR) family and catalyzes the reduction of the C15=C16 double bond, followed by the C4=C5 double bond of biliverdin IXα to produce phycourobilin. Among the diverse FDBR enzymes that catalyze site-specific reduction reactions of bilins, PubS lineage is the only one that reduces the C4=C5 double bond. This family can be broadly divided into four-electron reduction enzymes, which catalyze two successive two-electron reductions, such as PubS, and two-electron reduction enzymes, which catalyze a single two-electron reduction. The crystal structures of diverse FDBRs, excluding PubS, have unraveled that there are two distinct binding modes in the substrate-binding pocket. In this study, we focused on the arginine (Arg) residues that is considered crucial for substrate-binding mode in two-electron reduction enzymes. Through sequence alignment and comparison with the predicted structure of PubS, we identified a residue in PubS that corresponds to the Arg residue in the two-electron reduction enzymes. We further introduced mutations to avoid the steric hindrance associated with changes in the binding mode. Biochemical characterization of these variants showed that we successfully modified PubS from a four-electron reduction enzyme to a two-electron reduction enzyme with the accumulation of radicals. Our results provide insight into the molecular mechanisms of the chromophore binding mode and proton donation from acidic residues**.

## Introduction

**Fig. 1:**
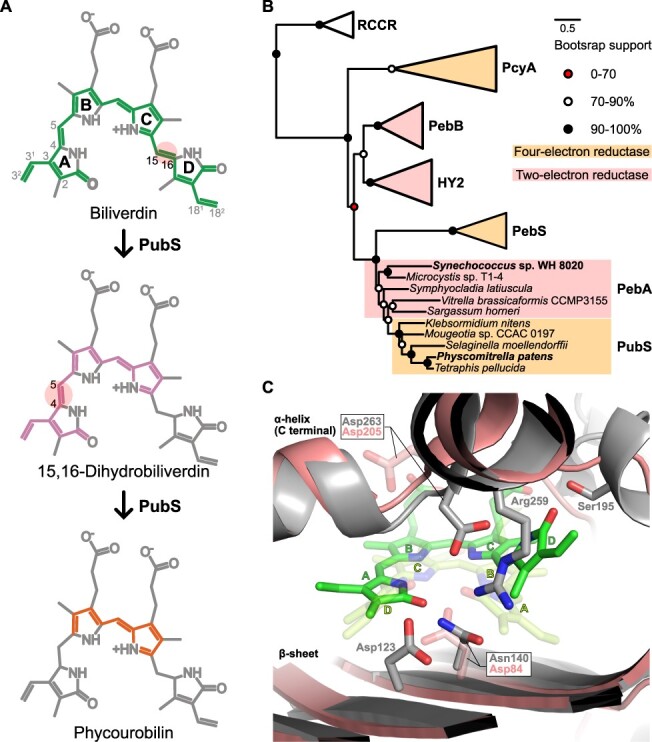
The biochemical, sequence and structural features of FDBRs focusing on PubS. (A). The PUB synthesis pathway by PubS. The transparent circle indicates the reduction site. The conjugated regions are highlighted with colors, which correspond to the colors of the chromophores themselves. (B) The ML phylogenetic tree of FDBRs with the RCCR sequences set as an outgroup. RCCR is the red chlorophyll catabolite reductase. (C) Close-up view of the BV-binding pocket in the crystal structures of PebA (salmon, PDB ID: 2X9O) derived from *Synechococcus* sp. WH 8020, with BV as the substrate (green), and HY2 (gray, PDB ID: 6KME) derived from *S. lycopersicum* with BV as the substrate (pale green).

Phycourobilin (PUB) is a type of bilin pigment and a yellow linear tetrapyrrole compound. In marine cyanobacteria, the phycobilisome, a light-harvesting complex for photosynthesis, often attaches blue-light absorbing PUB (*λ*_max_ 495 nm) in addition to the green-absorbing phycoerythrobilin (PEB) (*λ*_max_ 550 nm). They show a chromatic acclimation that regulates the ratio of phycobiliproteins attached to PUB according to the light environment (Palenik 2001, Everroad et al. 2006, Six et al. 2007, Sanfilippo et al. 2019). Previous studies on the chromatic acclimation mechanism indicated that MpeZ is a PEB lyase isomerase that attaches PEB to phycobiliprotein and isomerizes it to PUB (Shukla et al. 2012). In this case, PUB synthesis is strictly coupled to ligation to the phycobiliprotein without the production of free PUB chromophore. In contrast, PEB is synthesized by PebA and PebB or PebS, which belong to the ferredoxin-dependent bilin reductase (FDBR) family. Remarkably, in 2012, phycourobilin:ferredoxin oxidoreductase (PubS), which belongs to the FDBRs and synthesizes PUB from biliverdin (BV) via the intermediate 15,16-dihydrobiliverdin (15,16-DHBV), was discovered in non-vascular plants (**Fig. 1A**) (Chen et al. 2012). Unlike the lyase isomerase, a free PUB chromophore is produced. In non-vascular plants, it has been reported that PUB is involved in the response to photomorphogenesis via phytochromes (Chen et al. 2012). However, a mutational analysis of PubS has not been conducted.

The enzymes of the FDBR family exhibit a high reduction specificity for BV or 15,16-DHBV as substrates, including phycocyanobilin:ferredoxin oxidoreductase (PcyA, EC 1.3.7.5), 15,16-DHBV:ferredoxin oxidoreductase (PebA, EC 1.3.7.2), PEB:ferredoxin oxidoreductase (PebB, EC 1.3.7.3), phycoerythrobilin synthase (PebS, EC 1.3.7.6) and phytochromobilin synthase (HY2, EC 1.3.7.4) (Frankenberg et al. 2001, Kohchi et al. 2001, Dammeyer and Frankenberg-Dinkel 2006, Dammeyer et al. 2008a). Within the FDBR family, enzymes can be classified into two major groups: two-electron reduction enzymes (PebA, PebB and HY2) and four-electron reduction enzymes (PcyA and PebS). The phylogenetic analysis of the FDBR family, with the red chlorophyll catabolite reductase as the outgroup, revealed that HY2 and PebB are classified within the same clade, with a sister clade that contains PebS, PebA and PubS. PcyA is positioned as an outgroup of these clades (**Fig. 1B**) (Rockwell et al. 2023).

The X-ray crystal structure of all FDBRs bound to substrates, excluding PubS, indicates a single-domain architecture that folds into an α-helix/β-sheet/α-helix sandwich (Hagiwara et al. 2006, Dammeyer et al. 2008b, Busch et al. 2011, Sommerkamp et al. 2019, Sugishima et al. 2020). For each FDBR, the substrate is bound between the central β-sheet and α-helix on the C-terminus. Regarding the substrate-binding mode, for the FDBRs that use BV as a substrate, all except HY2 observed the *ZZZ*/all-*syn* (U-shaped) conformation when BV is bound. From the perspective of the C-terminal helix side, the rings are arranged in the order: D–C–B–A (**Fig. 1C**). On the other hand, in HY2, the orientation of BV is reversed compared with its configuration in other FDBRs, adopting an A–B–C–D arrangement. In addition, the D-ring in the inverted BV configuration is tilted at the C15–C16 single bond leading to a binding conformation known as *ZZ*-/*ss*- (**Fig. 1C**) (Sugishima et al. 2020). PebB, which uses 15,16-DHBV instead of BV as its substrate, exhibits a binding mode that is similar to the conformation of BV in HY2: A–B–C–D arrangement with a *ZZZ*/*ssa* conformation (Sommerkamp et al. 2019).

Based on the relationship between the reduction sites and binding modes of BV or 15,16-DHBV in each of the FDBRs, only HY2 and PebB, which catalyze reduction solely on the A-ring, arrange the rings of the substrate in the order A–B–C–D, with the D-ring being tilted in their binding (**Table 1**). It is suggested that these common features would be established by an arginine (Arg) residue that is highly conserved among HY2 and PebB (Sommerkamp et al. 2019, Sugishima et al. 2020). The Arg residue is oriented toward the inside of the substrate-binding pocket, and residues around the D-ring are replaced with smaller side chains to accommodate the tilted D-ring (**Fig. 1C**).

**Table 1:** The binding modes and reduction sites of the FDBRs

FDBRs	Conformation	Reduction site	Substrate	PDB ID
HY2	*ZZZ/ssa*	① 2,3,3^1^,3^2^-diene system of the A-ring	BV	6KME
PebB	*ZZ-/ss*- (tilted D-ring)	① 2,3,3^1^,3^2^-diene system of the A-ring	15,16-DHBV	6QX6
PebA	*ZZZ*/all-*syn*	① 15,16-double bound of the CD-ring	BV	2X9O
PcyA	*ZZZ*/all-*syn*	① 18^1^,18^2^-double bound of the D-ring ② 2,3,3^1^,3^2^-diene system of the A-ring	BV	2D1E
PebS	*ZZZ*/all-*syn*	① 18^1^,18^2^-double bound of the D-ring ② 2,3,3^1^,3^2^-diene system of the A-ring	BV	2VCK

In this study, for the first time, we conducted a mutational analysis of PubS, the only enzyme among the FDBRs that catalyzes the reduction of the double bond between the C- and D-rings and the reduction of the double bond between the A- and B-rings. Based on previous crystal structures, we successfully modified PubS from a four-electron reduction enzyme to a two-electron reduction enzyme by introducing two amino acid residues: an Arg residue and an alanine (Ala) residue.

## Results

### Sequence alignment and comparison of the FDBR structures

**Fig. 2:**
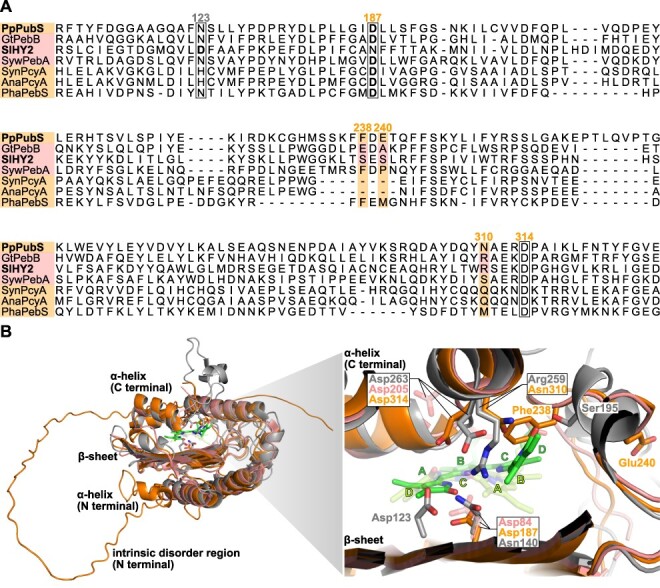
Multiple sequence alignments of the FDBRs and comparison of the FDBR crystal structures. (A) Comparison of the FDBR sequences. PpPubS, *Physcomitrella patens*; GtPebB, *Guillardia theta* CCMP2712; SlHY2, *Solanum lycopersicum*; SywPebA, *Synechococcus* sp. WH 8020; SynPcyA, *Synechocystis* sp. PCC 6803; AnaPcyA, *Anabaena* sp. PCC 7120; PhaPebS, cyanophage P-SSM2. (B) The overall structures are superimposed on the predicted structure of PpPubS (orange, AlphaFoldDB ID: AF-I3TC50-F1) with the crystal structures of SlHY2 (gray, PDB ID: 6KME) and SywPebA (salmon, PDB ID: 2X9O) (left). The expanded view of the substrate-binding pocket is shown (right). BV from PebA is positioned in the substrate-binding pocket.

We conducted an alignment analysis of amino acid sequences and structural comparisons to investigate the amino acid residue in PubS from *Physcomitrella patens* (PpPubS) that corresponds to the Arg-259 residue in HY2 from *Solanum lycopersicum* (SlHY2), which is highly conserved among HY2 and PebB and would be crucial for the unique chromophore binding mode. The crystal structures of PcyA from *Synechocystis* sp. PCC 6803 (SynPcyA) and *Anabaena* sp. PCC 7120 (AnaPcyA), PebA from *Synechococcus* sp. WH 8020 [SywPebA, Protein Data Bank (PDB) ID: 2X9O], PebS from cyanophage P-SSM2 (PhaPebS), PebB from *Guillardia theta* CCMP2712 (GtPebB), HY2 from *S. lycopersicum* (SlHY2, PDB ID: 6KME) and PubS from *P. patens* (PpPubS, AlphaFoldDB ID: AF-I3TC50-F1) were compared. Based on multiple sequence alignment of these sequences and structure comparison of the structure of PpPubS from AlphaFoldDB against the structure of SlHY2, we found that Asn-310 in PpPubS corresponds to Arg-259 in SlHY2 (**Fig. 2A, B**). In SlHY2, the highly conserved Ser-195 residue with a relatively small side chain, which provides sufficient structural space to accommodate the tilted D-ring of BV, corresponds to Glu-240 of PpPubS by multiple sequence alignment (**Fig. 2A**). In the predicted structure of PpPubS, however, the Glu-240 side chain was oriented opposite the BV. Comparison between the SlHY2 structure and the PpPubS predicted structure revealed that the Phe-238 residue in PpPubS occupied the space for the tilted D-ring located close to the Ser-195 residue and oriented toward the BV (**Fig. 2B**). In summary, Asn-310, Glu-240 and Phe-238 are candidates for mutagenesis targets to engineer the enzymatic properties of PpPubS.

### Introduction of an Arg residue into PpPubS

**Fig. 3:**
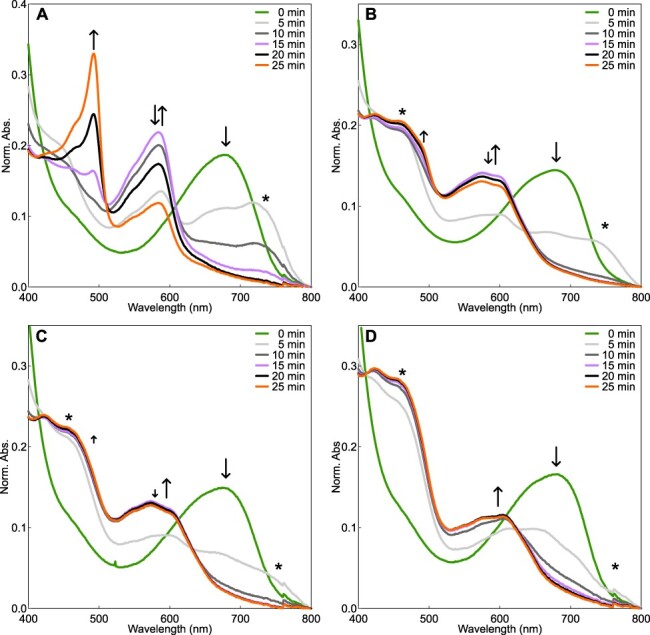
Enzymatic assay of PpPubS wild-type and variants observed using spectroscopy. Spectroscopic observations of the reaction mixtures containing (A) wild-type, (B) N310R, (C) N310R_E240A and (D) N310R_F238A at various time points. Upward and downward arrows indicate an increase and decrease in absorbance, respectively. The asterisks correspond to the radical components.

**Fig. 4:**
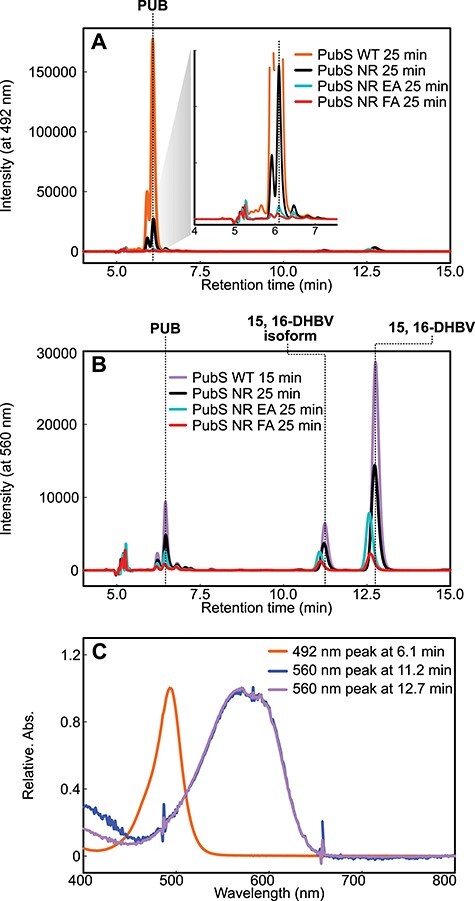
HPLC analysis of the final products in PpPubS wild-type and variants. (A) The chromatogram obtained at 492 nm and an expanded view of the peak detected at approximately 6 min are shown. (B) The chromatogram obtained at 560 nm is shown. (C) Absorption spectra showing the peaks of the reaction products at 6.1, 11.2 and 12.7 min.

The full-length sequence of PpPubS, which includes an intrinsic disorder region at the N-terminus (**Fig. 2B**), exhibited almost no enzymatic activity (data not shown). Therefore, we deleted the intrinsic disorder region from residues 1 to 55 (ΔΝ_PpPubS). Consistent with previous studies, the spectroscopic analysis of the wild-type ΔΝ_PpPubS showed a large decrease in the absorbance at 690 nm derived from BV at 10 min after NADPH addition, followed by an increase in the absorbance at 580 nm, which was likely derived from 15,16-DHBV. Subsequently, an increase in the absorbance at 492 nm, likely derived from PUB, was observed as the absorbance of 15,16-DHBV decreased (**Fig. 3A**). The increase in absorbance at 750 nm within 0–10 min corresponds to substrate radicals based on previous studies (Tu et al. 2004, Miyake et al. 2020, 2022). We further performed HPLC analysis of the wild-type samples at 15 and 25 min after NADPH addition. Based on previous studies and the absorption wavelength of each peak, we identified 15,16-DHBV and PUB based on 560 and 492 nm absorbances, respectively, in which the retention times were approximately 12.7 and 6.1 min, respectively (Chen et al. 2012, Sommerkamp et al. 2019) (**Fig. 4**). Furthermore, we assigned the peak with a retention time of approximately 11.2 min as an isoform of 15,16-DHBV, based on its absorption wavelength being similar to that of 15,16-DHBV (**Fig. 4C**). The substrate BV was detected at 31 min in the HPLC analysis (**Supplementary Fig. S1A**). We first replaced Asn-310 in ΔΝ_PpPubS with an Arg residue, which was oriented toward the center of BV and expected to affect the tilt of the D-ring in SlHY2. PpPubS_N310R exhibited a decrease in BV absorbance similar to the wild-type molecule at 10 min after NADPH addition. An absorption component corresponding to the intermediate 15,16-DHBV was concomitantly increased but displayed a broad absorption around 580–600 nm for the ΔΝ_PpPubS_N310R. This is in contrast to that observed for the wild-type molecule, which exhibited a sharp absorption peak at 580 nm. After that, ΔΝ_PpPubS_N310R showed a slight decrease in the absorption component between 580 and 600 nm and a slight increase in the absorption component around 490 nm (**Fig. 3B**). HPLC analysis of the final product from ΔΝ_PpPubS_N310R detected a small amount of PUB (**Fig. 4A**). Furthermore, peaks were observed at 11.2 and 12.7 min, albeit with slightly lower intensity compared with that of the wild-type (**Fig. 4B**). The substrate BV was barely detected (**Supplementary Fig. S1**).

### Handling the tilting of the pyrrole ring

**Fig. 5:**
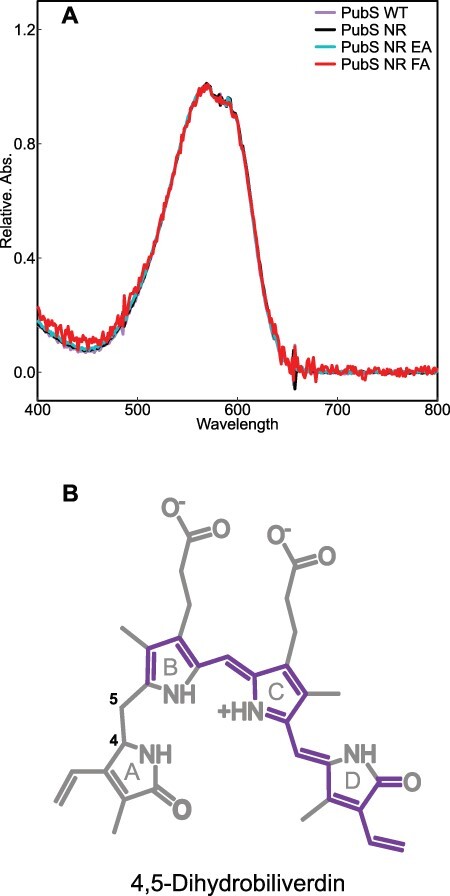
Potential bilin chromophores synthesized by PpPubS variants. (A) A comparison of the absorption wavelengths of the peak components by HPLC analysis was obtained at approximately 12.5 min in the chromatogram at 560 nm. (B) The expected structure of 4,5-DHBV, with the conjugated system is highlighted in color.

Because the introduced Arg residue resulted in the tilting of either the A-ring or D-ring of BV in PpPubS, the presence of the bulky Glu-240 or Phe-238 residues at the tilted position may cause steric hindrance, resulting in the instability of the tilted geometry. Therefore, we constructed two doubly mutated variants, ΔΝ_PpPubS_N310R_E240A and ΔΝ_PpPubS_N310R_F238A. Spectroscopic observations of these variants showed a decrease in BV after the addition of NADPH and an increase in the broad absorption component between 580 and 600 nm (**Fig. 3C, D**). After that, ΔΝ_PpPubS_N310R_E240A showed a slight decrease in the absorption component between 580 and 600 nm and an increase at 490 nm, whereas ΔΝ_PpPubS_N310R_F238A showed no change in the absorption component between 580 and 600 nm until 25 min (**Fig. 3C, D**). Consistent with these spectroscopic observations, HPLC analysis revealed that ΔΝ_PpPubS_N310R_E240A slightly detected PUB, whereas ΔΝ_PpPubS_N310R_F238A did not detect PUB. Moreover, both variants detected a peak faster compared with the peak of 15,16-DHBV in the wild-type (**Fig. 3D**). The peaks detected faster in ΔΝ_PpPubS_N310R_E240A and ΔΝ_PpPubS_N310R_F238A had the same absorption component as the peak for 15,16-DHBV detected in the wild-type (**Fig. 5A**). The substrate BV could not be detected for both ΔΝ_PpPubS_N310R_E240A and ΔΝ_PpPubS_N310R_F238A (**Supplementary Fig. S1**).

## Discussion

The crystal structures of FDBRs, excluding PubS, were previously determined for the substrate-free or substrate-binding form (Hagiwara et al. 2006, Dammeyer et al. 2008b, Busch et al. 2011, Sommerkamp et al. 2019, Sugishima et al. 2020). The FDBRs share a typical overall structure that features a sandwich of α-helix, β-sheet and α-helix, whereas the substrate-binding modes can be broadly divided into two categories. FDBRs other than HY2 and PebB bind to their substrate BV in a helical (*ZZZ*/all-*syn*) configuration (Hagiwara et al. 2006, Dammeyer et al. 2008b, Busch et al. 2011). In contrast, HY2 and PebB, which only catalyze a two-electron reduction on the A-ring, bind to their respective substrates, BV and 15,16-DHBV, in a flipped manner and with the tilted D-ring (Sommerkamp et al. 2019, Sugishima et al. 2020). Phylogenetically, PpPubS and its homologs form a single lineage with 15,16-DHBV synthase (PebA) (**Fig. 1B**). The PubS sequences were derived from a deep branch of the PebA sequences within the lineage. Overall, PpPubS would also bind to BV in a *ZZZ*/all-*syn* configuration in addition to PebA.

In this study, we successfully converted PpPubS, a four-electron reduction enzyme, into a two-electron reduction enzyme by substituting two amino acids. By comparing the absorption spectra of the BV complexes, we observed that all mutants exhibited a shoulder absorbance in the shorter wavelength region compared with the wild-type (**Supplementary Fig. S2**). A similar shoulder absorbance was also observed for HY2 and PebB, which bind to the substrate in *ZZZ*/*ssa* or *ZZ*-/*ss*- configurations (Sommerkamp et al. 2019, Sugishima et al. 2020). This suggests that introducing mutations changed the substrate-binding mode. We considered two possibilities for the linear tetrapyrrole chromophore synthesized by the ΔΝ_PpPubS_N310R_F238A and ΔΝ_PpPubS_N310R_E240A variants (**Figs. 2D** and **3B, D**). The first possibility is that the 15,16-DHBV may be synthesized as the final product. This is supported by the peak detected at approximately 12.7 min in both variants that exhibited the same absorption wavelength as 15,16-DHBV in the wild-type (**Figs. 4B** and **5A**). In this case, the introduction of Arg and Ala residues in ΔΝ_PpPubS would not cause BV flipping as observed in the binding mode of SlHY2, and tilting the A-ring causes the C4=C5 double bond between the A- and B-rings to move away from the reaction center aspartic acid (Asp) residue located on the β-sheet. This inhibits the reduction reaction of the double bond and only reduces the C15=C16 double bond. In contrast, when the PUB peak is considered as the internal control, HPLC analysis revealed peaks at slightly different retention times between the two double mutants and the wild-type (**Figs. 4B** and **5A**). In this situation, we may provide another possibility that the final product is a linear tetrapyrrole chromophore with a C4–C5 single bond and a C15=C16 double bond (**Fig. 1D**). Hereafter, this chromophore is called 4,5-dihydrobiliverdin (4,5-DHBV), which was named after the reduction site. Considering that BV is a nearly symmetrical chromophore, it is possible that the π-conjugated system length of 15,16-DHBV conjugated from the A-ring to the C-ring may be similar to that of 4,5-DHBV conjugated from the B-ring to the D-ring. This could potentially result in the same absorption properties (**Figs. 1A** and **5B**); however, slight differences in the side chains between the A- and D-rings of these two chromophores may result in slightly different physical properties. These differences may be responsible for the difference in retention times observed in the HPLC analysis. Thus, we assume that both the BV inversion and the D-ring tilting occur by mutation in a manner similar to SlHY2 as the second possibility. Overall, we could not conclusively determine the linear tetrapyrrole chromophore synthesized by the two-electron reduction enzyme.

Because the doubly mutated variants catalyze a two-electron reduction, the introduced Arg residue is considered to be oriented toward the substrate. In SlHY2 and GtPebB, the Arg residue is oriented toward the substrate to form a salt bridge with the Asp residue on the α-helix side (**Fig. 1C**). For the predicted structure of PpPubS wild-type, the corresponding Asp residue on the α-helix side (Asp-314) is oriented toward the substrate, similar to SlHY2 (**Fig. 2B**). Thus, the side chain of the introduced Arg residue would be stably fixed toward the substrate by forming a salt bridge with Asp-314. In the case of SlHY2, the Asp-263 residue, corresponding to Asp-314 of PpPubS, and the Asp-123 residue located on the β-sheet are considered to function as proton donors to the substrate (Sugishima et al. 2020). Therefore, a larger absorption component corresponding to the radical in ΔΝ_PpPubS_N310R than the wild-type (**Fig. 3**, asterisks) may be due to the arranged configuration of Asp-314 that is slightly away from the substrate affected by the salt bridge with the introduced Arg residue, which prevents second proton donation and increasing radicals (Tu et al. 2004, Stoll et al. 2009, Busch et al. 2011). In addition, for the double mutants, Glu-240 or Phe-238, which may cause steric hindrance to the tilted pyrrole ring, is replaced with an Ala residue, and the resulting absorption component corresponding to the radical further increased more than in ΔΝ_PpPubS_N310R (**Fig. 3**, asterisks). This may be attributed to the resolution of steric hindrance, enabling a flexible tilted pyrrole ring and affecting the configuration of Asp-314 to a larger extent. In fact, in SlHY2 and GtPebB, where the D-ring is tilted, highly conserved Ser or Gln residues around the D-ring form hydrogen bonds with the lactam oxygen atom of the D-ring, respectively, to stabilize the tilted D-ring (Sommerkamp et al. 2019, Sugishima et al. 2020). The introduction of polar uncharged amino acids in double mutants of PpPubS around the lactam oxygen atom of the tilted pyrrole ring may promote efficient second proton donation. Further engineering would be facilitated by determining the PpPubS structure.

## Materials and Methods

### Bacterial strains and growth media

The *Escherichia coli* strain JM109 was used for plasmid construction, while the *E. coli* strain C41 was used for protein expression. *Escherichia coli* cells were grown in Luria–Bertani (LB) medium containing appropriate antibiotics. For protein expression, the cells were grown in LB medium at 37°C until an optical density at 600 nm reached 0.4–0.8. Isopropyl-β-d-1-thiogalactopyranoside was added at a final concentration of 0.1 mM. Subsequently, the cells were cultured at 18°C overnight, followed by centrifugation to collect the cell pellets.

### Bioinformatics

Nucleotide and amino acid sequences of the *P. patens* proteins were obtained from the UniProt Database (UniProt Consortium, 2023). Multiple alignments of the FDBR sequences were constructed using MAFFT v7.505 (--genafpair --maxiterate 1000) (Katoh and Standley 2013). To construct a phylogenetic tree, we used trimAl v1.4.1 (-gappyout) (Capella-Gutiérrez et al. 2009) and IQ-TREE v2.2.2.7 (-bb 1000, -alrt 1000) for maximum likelihood (ML) phylogenetic analysis (Hoang et al. 2018, Minh et al. 2020). To select the substitution model, we used IQ-TREE’s built-in ModelFinder and selected Q.pfam + I + G4, which had a minimized Bayesian information criterion score among 1,232 models. Statistical robustness was assessed using the ultrafast bootstrap value. For structural comparison, the Pairwise Structure Comparison tool from the RCSB PDB website was used (Burley et al. 2022).

### Plasmid construction

Plasmids for protein expression in *E. coli* were constructed by inserting the PubS gene fused with a His-tag sequence on its N-terminus into the pET28a vector. The gene fragment was artificially synthesized (Azenta Life Sciences, South Plainfield, NJ), and the codon was optimized for expression in *E. coli*. The PrimeSTAR Max Basal Mutagenesis kit (Takara Bio Inc., Kusatsu, Shiga, Japan) with appropriate nucleotide primers was used to perform site-directed mutagenesis of PubS (**Supplementary Table. S2**). The expression constructs were verified by nucleotide sequencing.

### Protein expression and purification

The PubS wild-type and variant proteins were expressed in *E. coli* C41. Cell culture volumes were in the range of 1–2.5 l. Cells were disrupted in lysis buffer containing 20 mM HEPES-NaOH, pH 7.5, 0.1 M NaCl and 10% (w/v) glycerol by three passages through an Emulsiflex C5 high-pressure homogenizer at 12,000 psi (Avestin, Inc., Ottawa, Ontario, Canada). The mixtures were centrifuged at 165,000×*g* for 30 min, and the supernatants were filtered through a 0.2-μm cellulose ether membrane before loading onto a nickel-affinity His-trap column (GE Healthcare, Chicago, IL) using an ÄKTAprime plus unit (GE Healthcare). The column was washed with lysis buffer containing 100 mM imidazole, and the His-tagged proteins were eluted in a linear gradient of lysis buffer containing 100–400 mM imidazole (1 ml/min, total 15 min). After incubation with 1 mM EDTA for 1 h, the proteins were dialyzed against lysis buffer with or without 1 mM dithiothreitol to remove EDTA and imidazole. The Bradford assay (Bio-Rad Laboratories, Hercules, CA) was performed to measure protein concentration using bovine serum albumin (BSA) to generate a standard curve.

### Anaerobic bilin reductase assay

All solutions were deaerated by N_2_ bubbling. The reaction mixture contained 10 μM PubS, 5 μM ferredoxin from cyanobacterium *Synechococcus* sp. PCC 7002, 15 nM ferredoxin:NADP^+^ oxidoreductase from cyanobacterium *Synechocystis* sp. PCC 6803, 100 mM KCl, 10 μM BSA and 10 μM BV in 25 mM TES-KOH (pH 8.5). The reaction mixture was placed in a septum-stoppered quartz cuvette, and 99% pure N_2_ gas was passed for 30 min, followed by incubation at 30°C for 30 min. To control the reaction temperature while simultaneously stirring it, we used a spectrophotometer equipped with a thermoelectric single-cell holder (S-1700, Shimadzu Corporation, Kyoto, Japan). PubS activity was initiated by injecting NADPH (100 μM final concentration) into the reaction mixture in cuvettes using a gas-tight syringe. The reaction was terminated by adding 1 ml of 0.1% trifluoroacetic acid (TFA) and incubating the reaction mixture on ice. The enzymatic products were analyzed by spectroscopy and HPLC.

### HPLC analysis

Bilin reductase assay mixtures were loaded onto Sep-Pak C18 cartridges (Waters Corporation, Milford, MA) preconditioned by performing a 3-ml wash with acetonitrile to wet the Sep-Pak, a 3-ml wash with MilliQ water and a 3-ml wash with 0.1% (v/v) TFA. After the sample was loaded onto the Sep-Pak, it was washed with 3 ml of 0.1% (v/v) TFA and 100% acetonitrile/0.1% (v/v) TFA (20:80). The bilin products were eluted from the Sep-Pak with 1 ml of 100% acetonitrile. The eluate was passed through a 0.2-µm polytetrafluoroethylene membrane filter and then dried using a Speed-Vac evaporator. Samples were first dissolved in 20 µl of dimethyl sulfoxide and then diluted with 100 µl of the HPLC mobile phase (45:55 v/v acetone:20 mM formic acid). After the samples were dissolved, they were loaded onto the HPLC system (Shimadzu Corporation, Kyoto, Japan) using a reverse-phase HPLC column (InertSustainSwift C18, GL Sciences Inc., Tokyo, Japan). For each sample, 10 µl was injected into the HPLC system and monitored at 380 nm. The mobile phase was acetone:20 mM formic acid [45:55 (v/v)]. The flow rate was set at 0.6 ml/min.

## Supplementary Material

pcae098_Supp

## Data Availability

The data underlying this study are available in the article and **supplementary data**.
