# Supplementary material for: Engineering of Phycourobilin Synthase: PubS to a Two-Electron Reductase
Source: Plant Cell Physiol. 2024 Sep 4;66(2):229–37. doi: 10.1093/pcp/pcae098 (PMC11879140; doi:10.1093/pcp/pcae098)
Supplement: pcae098_Supp [file pcae098_supp.zip › suppl_data/pcp-2024-e-00079-File007.pdf]

Table S1. Primer used in the study.

Name		Sequence
PubS_N310R	Fw	5'-CAATATCGTGCGGAACGTGATCCGG-3'
	Rv	5'-AGGACGCGTACGATCAATATCGTGCGGAA-3'
PubS_N310R_E240A	Fw	5'-TTTGATGCGACCCAGTTCTTTAGCAAATACCTGATCT-3'
	Rv	5'-GTCACATGAGCAGCAAGTTCTTTGATGCGACCCAG-3'
PubS_N310R_F238A	Fw	5'-AAGTTCGCGGATGAAACCCAGTTCTTTAGCAAATACCTG-3'
	Rv	5'-CGGTCACATGAGCAGCAAGTTCGCGGATGAA-3'

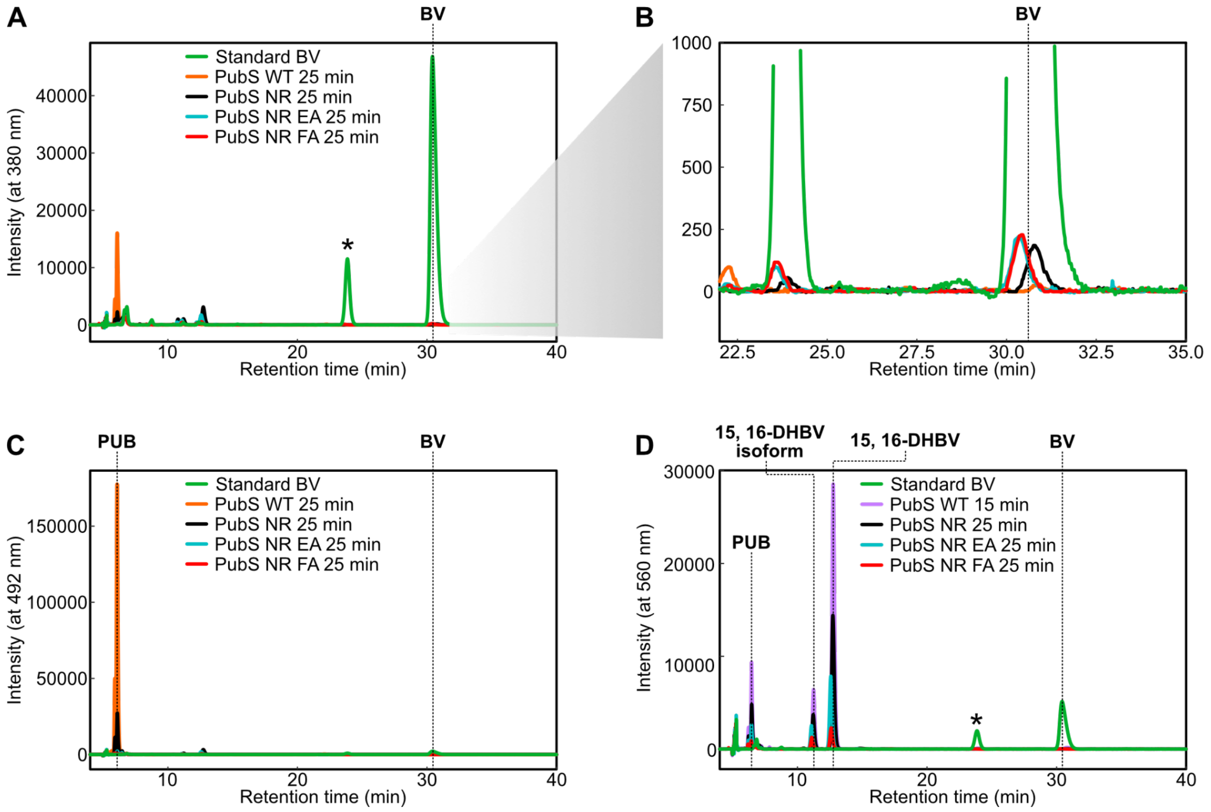

Fig. S1. Full-time HPLC chromatography. The chromatogram was obtained at (A) 380 nm, (C) 492 nm, and (D) 560 nm. (B) The expanded view of (A) between 22.5 and 35 min is shown. The asterisks correspond to contamination.

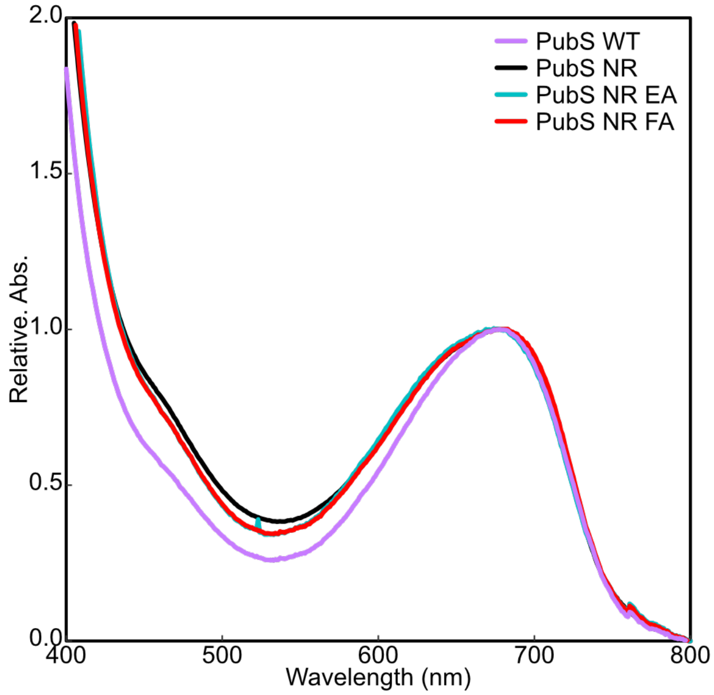

Fig. S2. The absorption spectra of the substrate BV complex were overlaid for the wild-type and all of the mutants. Each spectrum normalized to λ_{max} of 600-700 nm.
